# In pursuit of certainty: can the systematic review process deliver?

**DOI:** 10.1186/1472-6947-13-25

**Published:** 2013-02-20

**Authors:** Deborah Saltman, Debra Jackson, Phillip J Newton, Patricia M Davidson

**Affiliations:** 1 Faculty of Health, University of Technology Sydney (UTS), Australia; 2 Department of Primary Care and Public Health, School of Public Health, Imperial College, London, UK

**Keywords:** Systematic review, Research-in-practice, Research implementation, Translational research, Evidence-based practice, Clinical decision-making

## Abstract

**Background:**

There has been increasing emphasis on evidence-based approaches to improve patient outcomes through rigorous, standardised and well-validated approaches. Clinical guidelines drive this process and are largely developed based on the findings of systematic reviews (SRs). This paper presents a discussion of the SR process in providing decisive information to shape and guide clinical practice, using a purpose-built review database: the Cochrane reviews; and focussing on a highly prevalent medical condition: hypertension.

**Methods:**

We searched the Cochrane database and identified 25 relevant SRs incorporating 443 clinical trials. Reviews with the terms ‘blood pressure’ or ‘hypertension’ in the title were included. Once selected for inclusion, the abstracts were assessed independently by two authors for their capacity to inform and influence clinical decision-making. The inclusions were independently audited by a third author.

**Results:**

Of the 25 SRs that formed the sample, 12 provided conclusive findings to inform a particular treatment pathway. The evidence-based approaches offer the promise of assisting clinical decision-making through clarity, but in the case of management of blood pressure, half of the SRs in our sample highlight gaps in evidence and methodological limitations. Thirteen reviews were inconclusive, and eight, including four of the 12 conclusive SRs, noted the lack of adequate reporting of potential adverse effects or incidence of harm.

**Conclusions:**

These findings emphasise the importance of distillation, interpretation and synthesis of information to assist clinicians. This study questions the utility of evidence-based approaches as a uni-dimensional approach to improving clinical care and underscores the importance of standardised approaches to include adverse events, incidence of harm, patient’s needs and preferences and clinician’s expertise and discretion.

## Background

Much has been written about evidence-based medicine (EBM) and its potential to inform practice. The terminology surrounding the aggregation of evidence in a systematic way implies that there is a practical import to this work, and the terms “evidence-based practice” (EBP) and “evidence-based approaches” (EBA) have arisen from this concept. The EBA are among the most significant contemporary ways of conceptualising health care, both theoretically and practically [1], and are viewed by advocates as the favoured means of integrating research findings into clinical practice [2].

Despite the appeal of EBA, and the widespread recognition of this approach in driving care, they present a number of challenges, and there has been a steady undercurrent of concern about their pervasive acceptance and promotion. Debate has focussed on several key factors, including the nature of evidence, particularly the privileging of some forms of evidence and the marginalising and devaluing of others [2-5]. Concerns that the domination of the EBA has (and will continue to) foster ‘a very rational, traditional, biomedical approach to research use/evidence-based practice’ [5] forms a crucial plank of the critique. Additional major critiques of EBA highlight concerns that they do not meet the challenges of rare diseases or unusual presentations, and may foster a “cookbook” approach to practice [6]. An additional view is that they may potentially have a deleterious effect on patient/physician relationships; including the charge that they create or contribute to an environment of paternalism and reductionism that can render patient preference and experience invisible and irrelevant [1-3,7]. According to this critique, EBA can foster a therapeutic milieu in which patients are positioned as passive recipients in the medical encounter [1], and clinicians are limited in their ability to draw on intuitive and other forms of knowledge [7].

Notwithstanding the theoretical critique however, the aim of EBA is to facilitate the integration of the best available research evidence into practice by transparently assimilating information and aggregating data in ways that can help guide clinical decision-making [6]. Indeed, the EBA are attractive because they promote the idea of research-in-practice, yet present an alternative to each clinician locating, reading and assimilating the plethora of contemporary research literature on any clinical issue [6]. However, we argue that in some areas, even where a substantial body of literature exists, there is a lack of evidence to demonstrate that the EBA in fact provide the information necessary to effectively guide clinicians.

### Purpose of this paper

The purpose of this paper is to explore the efficacy of the systematic review (SR) process in providing decisive information to shape and guide clinical practice. To do this we examined SRs on the management of blood pressure. We chose this because hypertension is a well-researched, common and persistent condition with a significant effect on health outcomes [8]. The Cochrane reviews on reducing blood pressure became the lens through which we explored the efficacy of the SR process in providing contemporary and clear information upon which clinical practice decisions could be made.

## Methods

Independent manual searches of the reviews published on the *Cochrane Database of Systematic Reviews* (http://www.cochrane.org/cochrane-reviews) on 1/12/11 were undertaken to search for SRs on interventions to lower blood pressure. Reviews with the terms ‘blood pressure’ or ‘hypertension’ in the title were included. Once selected for inclusion, the abstracts were assessed independently by two authors. SRs were excluded if they pertained to blood pressure targets, pulmonary hypertension, hypertensive emergencies, acute cardiac events, gestational, postpartum, infant, neonatal and child hypertension. Furthermore, SRs were also excluded if the details of the number of clinical trials were not provided, and if the SR did not include any completed studies. Inclusions were independently audited by a third author.

## Results

Twenty-five SRs representing a combined total of 443 clinical trials were identified (see Table 1). Of these SRs, the majority reported pharmaceutical-based interventions. Salt intake, weight-reducing diets, timing of medication and relaxation therapies were each the subject of a single SR. Twelve SRs made conclusive assertions, that is, they used language that denoted a degree of certainty. The remaining SRs (n = 13) were inconclusive (see Table 2).

**Table 1:** Included systematic reviews

**Title**	**Date**	**Authors**	**No of trials**
Creatine and creatine analogues in hypertension and cardiovascular disease	2011	Horjus, et al.	11
Evening versus morning dosing regimen drug therapy for hypertension	2011	Zhao P, et al.	21
Long-term effects of weight-reducing diets in hypertensive patients	2011	Siebenhofer, et al.	8
Blood pressure lowering efficacy of beta-blockers as second-line therapy for primary hypertension	2010	Chen, et al.	20
Spironolactone for hypertension	2010	Batterink, et al.	5
Blood pressure lowering efficacy of potassium-sparing diuretics (that block the epithelial sodium channel) for primary hypertension	2010	Heran, et al.	6
Pharmacotherapy for hypertension in the elderly	2010	Musini, et al.	15
Blood pressure lowering efficacy of alpha blockers for primary hypertension	2009	Heran, et al.	10
Blood pressure lowering efficacy of angiotensin converting enzyme (ACE) inhibitors for primary hypertension	2009	Heran, et al.	92
Blood pressure lowering efficacy of angiotensin receptor blockers for primary hypertension	2009	Heran, et al.	46
Blood pressure lowering efficacy of coenzyme Q10 for primary hypertension	2009	Ho, et al.	3
Blood pressure lowering efficacy of diuretics as second-line therapy for primary hypertension	2009	Chen, et al.	56
Blood pressure lowering efficacy of loop diuretics for primary hypertension	2009	Musini, et al.	9
Blood pressure lowering efficacy of reserpine for primary hypertension	2009	Shamon & Perez	4
Blood pressure lowering in patients without prior cerebrovascular disease for prevention of cognitive impairment and dementia	2009	McGuinness, et al.	4
Methyldopa for primary hypertension	2009	Mah, et al.	12
Blood pressure lowering efficacy of renin inhibitors for primary hypertension	2009	Musini, et al.	6
First-line drugs for hypertension	2009	Wright & Musini	24
Relaxation therapies for the management of primary hypertension in adults	2009	Dickinson, et al.	25
Potassium supplementation for the management of primary hypertension in adults	2009	Dickinson, et al.	5
Magnesium supplementation for the management of primary hypertension in adults	2009	Dickinson, et al.	12
Calcium supplementation for the management of primary hypertension in adults	2009	Dickinson, et al.	13
Beta-blockers for hypertension	2009	Wiysonge, et al.	13
Combined calcium, magnesium and potassium supplementation for the management of primary hypertension in adults	2009	Beyer, et al.	3
Effect of longer-term modest salt reduction on blood pressure	2008	He & MacGregor	20
**TOTAL**			443

**Table 2:** Authors’ conclusions

**Authors**	**Comments from author’s conclusions (all comments taken directly from author conclusions as appeared on Cochrane Database of Systematic Reviews 1/12/11)**
Horjus, et al.	*This review found inconclusive evidence to decide on the use of creatine analogues in clinical practice.*
	*The effect on death and the size of myocardial infarction and the heart function were unclear.*
	Given the small sample size of the discussed trials and the heterogeneity of the population included in these reports, larger clinical studies are needed to confirm these observations.
Zhao P, et al.	In terms of BP lowering efficacy, for 24-hour SBP and DBP, the data suggests that better blood pressure control was achieved with bedtime dosing than morning administration of antihypertensive medication, the clinical significance of which is not known.
Siebenhofer, et al.	*The magnitude of the effects are uncertain as a result of the small number of patients and studies that could be included in the analyses.*
	*It is not known whether weight loss reduces mortality and morbidity.*
	No useful information on adverse effects was reported in the relevant trials.
	In conclusion, there is no evidence for effects of weight loss diets on death or long-term complications and adverse events.
	*In addition, results on blood pressure and body weight could be considered uncertain, because not all studies were included in the analyses.*
Chen, et al.	The different effect on diastolic BP means that beta-blockers have little or no effect on pulse pressure whereas thiazides cause a significant dose-related decrease in pulse pressure.
Batterink, et al.	From the limited available evidence, spironolactone appears to lower blood pressure compared to placebo to a similar degree in patients with primary (essential) hypertension when doses of 100-500 mg/day are given.
	A dose of 25 mg/day did not statistically significantly reduce systolic or diastolic blood pressure, compared to placebo.
	Given the lack of a dose-response, coupled with a possible increased risk in adverse events with higher doses, doses of 25 to 100 mg/day are reasonable.There is no evidence of the effect of spironolactone on clinical outcomes in hypertensive patients.
Heran, et al.	*ENaC blockers do not have a statistically or clinically significant BP lowering effect at low doses but trials at higher doses are not available.*
	The review did not provide a good estimate of the incidence of harms associated with ENaC blockers
Wiysonge, et al.	Thirteen RCTs were found and these trials suggested that first-line beta-blockers for elevated blood pressure were not as good at decreasing mortality and morbidity as other classes of drugs: thiazides, calcium channel blockers, and renin angiotensin system inhibitors.
Heran, et al.	The BP lowering effect of alpha blockers is modest; the estimate of the magnitude of trough BP lowering of -8/-5 mmHg is likely an overestimate.
	There are no clinically meaningful BP lowering differences between different alpha blockers.
	The review did not provide a good estimate of the incidence of harms associated with alpha blockers because of the short duration of the trials and the lack of reporting of adverse effects in many of the trials.
Heran, et al.	There are no clinically meaningful BP lowering differences between different ACE inhibitors.
	The BP lowering effect of ACE inhibitors is modest.
	The review did not provide a good estimate of the incidence of harms associated with ACE inhibitors because of the short duration of the trials and the lack of reporting of adverse effects in many of the trials
Heran, et al.	The evidence from this review suggests that there are no clinically meaningful BP lowering differences between available ARBs.
	The BP lowering effect of ARBs is modest and similar to ACE inhibitors as a class.
	The review did not provide a good estimate of the incidence of harms associated with ARBs because of the short duration of the trials and the lack of reporting of adverse effects in many of the trials
Ho, et al.	*Due to the possible unreliability of some of the included studies, it is uncertain whether or not coenzyme Q10 reduces blood pressure in the long-term management of primary hypertension*
Chen, et al.	Thiazides when given as a second-line drug have a dose related effect to lower blood pressure that is similar to when they are added as a first-line drug. This means that the BP lowering effect of thiazides is additive.
	Because of the short duration of the trials and lack of reporting of adverse events, this review does not provide a good estimate of the incidence of adverse effects of diuretics given as a second-line drug
Musini, et al.	*Based on the limited number of published RCTs, the SBP/DBP lowering effect of loop diuretics is modest … and is likely an overestimate due to the high risk of bias in the included studies.*
	There is no clinically meaningful BP lowering differences between different drugs within the loop diuretic class.
	*The dose ranging effects of loop diuretics could not be evaluated.*
	The review did not provide a good estimate of the incidence of harms associated because of the short duration of the trials and the lack of reporting of adverse effects in many of the trials.
Shamon & Perez	Reserpine is effective in reducing SBP roughly to the same degree as other first-line antihypertensive drugs.
	*However, we could not make definite conclusions regarding the dose-response pattern because of the small number of included trials.*
	More RCTs are needed to assess the effects of reserpine on blood pressure and to determine the dose-related safety profile before the role of this drug in the treatment of primary hypertension can be established
McGuinness, et al.	There is no convincing evidence from the trials identified that blood pressure lowering in late-life prevents the development of dementia or cognitive impairment in hypertensive patients with no apparent prior cerebrovascular disease.
	*There were significant problems identified with analysing the data, however, due to the number of patients lost to follow-up and the number of placebo patients who received active treatment. This introduced bias*.
	More robust results may be obtained by conducting a meta-analysis using individual patient data.
Mah, et al.	Methyldopa lowers blood pressure to varying degrees compared to placebo for patients with primary hypertension.
	*Its effect on clinical outcomes, however, remains uncertain.*
	Overall reporting of adverse effects was poor so no conclusions can be drawn about the adverse effect profile.
	This meta-analysis shows that methyldopa reduces systolic/diastolic blood pressure by approximately 13/8 mmHg compared to placebo.
Musini, et al.	Aliskiren has a dose-related blood pressure lowering effect better than placebo.
	This effect is similar to that determined for ACE inhibitors and ARBs
Wright & Musini	First-line low-dose thiazides reduce all morbidity and mortality outcomes.
	First-line ACE inhibitors and calcium channel blockers may be similarly effective but the evidence is less robust.
Dickinson, et al.	*In view of the poor quality of included trials and unexplained variation between trials, the evidence in favour of causal association between relaxation and blood pressure reduction is weak.*
	*It was difficult to disentangle their effects, especially as many trials used a combination of methods*
	*some of the reduction in blood pressure was almost certainly due to aspects of treatment that were not related to relaxation, such as frequent contact with professionals who were trying to help.*
Dickinson, et al.	*Due to small number of participants in the two high quality trials, the short duration of follow-up, and the unexplained heterogeneity between trials, the evidence about the effect of potassium supplementation on blood pressure is not conclusive.*
	Further high quality RCTs of longer duration are required to clarify whether potassium supplementation can reduce blood pressure and improve health outcomes.
	*Most included trials were of poor quality, so their results may not be reliable.*
	*The trials were not long enough or large enough to measure whether potassium supplements reduce the risk of death, heart attack or stroke, which may be caused by high blood pressure.*
	This review does not confirm whether potassium supplements can lower high blood pressure and therefore does not recommend them for treating hypertension.
	More trials enrolling a large number of participants with long periods of follow-up are necessary to know whether or not potassium supplements can lower high blood pressure.
Dickinson, et al.	*In view of the poor quality of included trials and the heterogeneity between trials, the evidence in favour of a causal association between magnesium supplementation and blood pressure reduction is weak and is probably due to bias.*
	*This is because poor quality studies generally tend to over-estimate the effects of treatment.*
	Larger, longer duration and better quality double-blind placebo controlled trials are needed to assess the effect of magnesium supplementation on blood pressure and cardiovascular outcomes.
Dickinson, et al.	*Due to poor quality of included trials and heterogeneity between trials, the evidence in favour of causal association between calcium supplementation and blood pressure reduction is weak and is probably due to bias.*
	This is because poor quality studies generally tend to over-estimate the effects of treatment.
	Larger, longer duration and better quality double-blind placebo controlled trials are needed to assess the effect of calcium supplementation on blood pressure and cardiovascular outcomes.
Musini, et al.	Treating healthy persons (60 years or older) with moderate to severe systolic and/or diastolic hypertension reduces all cause mortality and cardiovascular morbidity and mortality. The decrease in all cause mortality was limited to persons 60 to 80 years of age.
Beyer, et al.	*None of the trials were of high quality, so their results may not be reliable.*
	*We found no robust evidence that supplements of any combination of potassium, magnesium or calcium reduce mortality, morbidity or BP in adults*.
	More trials are needed to investigate whether the combination of potassium & magnesium is effective.
He & MacGregor	Our meta-analysis demonstrates that a modest reduction in salt intake for a duration of 4 or more weeks has a significant and, from a population viewpoint, important effect on blood pressure.
	These results support other evidence suggesting that a modest and long-term reduction in population salt intake could reduce strokes, heart attacks, and heart failure.

Key

*Italicised text* Inconclusive result.

Underlined text No/poor reporting of adverse effects or incidences of harm.

## Discussion

In eight SRs, including four of the 12 SRs that were considered to be conclusive, the lack of adequate reporting of potential adverse effects or incidence of harm was specifically noted (see Table 2). These factors would exert an influence on clinicians and on the confidence they can have in selecting specific treatments as a result of any SR. There has long been concern that clinicians can be slow to take up research findings into their practice, with a common focus being on accessibility of current information and the most efficacious means of dissemination [9,10]. However, findings from this exercise suggest there needs to be more of an onus on researchers to produce findings that are able to inform practice in a more useful way.

Attention is also drawn to the use and effect of extraneous words such as ‘appears to’ in reaching conclusions. It could be seen that use of this ambiguous language means that results are being presented cautiously but it could also have the effect of creating doubt or suggest an unwillingness or inability to have an opinion either way. Language is a problematic area in research [11], but much of the scrutiny about language and how it is used to present research focuses on the use of jargon or exclusionary language. When considering the use of ambiguous language - language which creates doubt and jeopardises meaning, questions about the nature of language and how it is used arise. How essential is the word (that causes the doubt) to the significance and meaning of the message? If we took those words out, would we have clear cut messages? If the words create the doubt, what is the point in having this process? If results cannot be interpreted with confidence, how can conclusions be reached through the SR process?

The inconclusive nature of several SRs raises a number of additional issues for consideration. The point of EBA as a systematic transparent process is to allow for the aggregation of data so that statistical significance may be achieved. In our sample, it was noted that some of the included studies were unreliable because of potential bias, questionable rigour or other factors which meant studies were deemed to be poor quality. The EBA have been the catalyst for the development of a ranking system, known as the hierarchy of evidence [12,13].

Darlenski et al. [6] make the point that good clinicians will be able to draw on EBA, and we concur with this, even where findings are inclusive. Furthermore, it is recognised and understood that information drawn from EBA is only one of a number of factors that inform clinical judgement. But, clearly there is a need to provide some guidance about interventions that may never be able to be proven in the current evidentiary way. At the moment this is left to institutions through mechanisms such as clinical pathways, regulators such as the National Institute of Clinical Excellence (NICE), and the National Health and Medical Research Council. Some progress is being made as groups both in the community and within organisations are trying to develop methodologies to assist this process, such as through modelling.

Systematic reviews are only as good as the sum of the parts. Strategies such as standardised reporting of clinical trials through CONSORT methods offer some promise in increasing the rigour and usefulness of research (http://www.consort-statement.org/). The recognition of complex interventions in clinical care is another important consideration [14]. In this instance some interventions may have high internal validity, yet less external validity.

## Conclusions

Though the EBA offer the promise of direction through clarity, in the case of reducing blood pressure, and despite the enormous amount of research that has been undertaken, the clarity offers little direction for the future, in the main, aggregating the past. Rather, it highlights evidentiary gaps and weaknesses. Indeed, one of the real values of the SR process is that it summarises what we know, which is potentially not what we need to know – what should I do for the 85 year old women sitting in my clinic?

Our results highlight a potential lack of efficacy of the SR process in driving future practice, which may leave future practice potentially open to other, less evidence-based directions. Krumholz [15] has recently highlighted the imperative of method in generating clinical research that is able to effectively inform practice. Through this exercise we have shown that even in areas where it might be thought to be strong, such as in the aggregation of drug trials, the results were often not clear and therefore their utility for informing practice is compromised. This may be a factor of the analytic techniques that are used. To be useful in clinical practice, there is a need to develop more robust methodologies and to ensure that research processes are well described and sufficiently transparent to allow data aggregation to occur with confidence.

Furthermore, it was noted that in some SRs inadequate information about prevalence of harm, complications and potential adverse effects were presented. In order that results are as clinically meaningful and useful as possible, there is a need to ensure reporting of presence or absence of these factors.

This review also emphasises the need for expertise and interpretation when developing clinical practice guidelines, particularly in the absence of robust Level 1 evidence and differences in clinical trial populations and patient groups. Indeed, it is certainly important to identify where the evidence does not support a particular line of practice. We argue that the corollary is not disproven by this approach, that is, practice may be improved by a particular intervention despite the null hypothesis, but the evidence isn’t strong enough in a normative way. There are many possible reasons why the level of evidence to support EBA in practice will not always be strong enough to inform practice. Assessing change in practice is difficult and robust methodologies have yet to be derived; the process is long-term and it will take some time for robust evidence to emerge; and, the evidence provided to support clinical decision-making may no longer be useful in practical settings. Based upon this review, data from SRs are only one part of the picture. Using systematic guideline development processes such as in ADAPTE framework are an important part of this process (http://www.adapte.org/www/).

Clearly there is a desire for contemporary and rigorous information upon which clinical treatment can be guided and individual clinical decisions made. The demand for this is evidenced in Cochrane’s own usage data, which states that “every day someone, somewhere searches The Cochrane Library every second, reads an abstract every two seconds and downloads a full-text article every three seconds” (http://www.cochrane.org: accessed 16/1/12).

Initially, the EBA have been viewed as a way of incorporating research into practice. The EBA does not always have high utility and reference for consistently assisting clinicians, particularly in groups not included in clinical trials. While the use of a single health issue may be viewed as a limitation of this paper; in using this common health issue – blood pressure management - as a lens, we have raised questions about whether SRs as the core of clinical guideline development can reliably influence practice in an interventional way, and about the strength of evidence that is available to inform clinical practice in some key areas.

## Competing interests

The authors declare that they have no competing interests.

## Authors’ contributions

DS conceptualized the project, analysed the data, and contributed to literature and internet searches and drafted parts of the manuscript. DJ analysed the data, and contributed to literature and internet searches and drafted parts of the manuscript. PN audited the data analysis, contributed to literature and internet searches and drafted parts of the manuscript. PMD reviewed the data analysis, contributed to literature and internet searches and drafted parts of the manuscript. DS, DJ, PN, PMD critically reviewed and revised many versions of the drafted manuscript. All authors read and approved the final manuscript.

## Pre-publication history

The pre-publication history for this paper can be accessed here:

http://www.biomedcentral.com/1472-6947/13/25/prepub
